# Reciprocal Interactions between Breast Tumor and Its Adipose Microenvironment Based on a 3D Adipose Equivalent Model

**DOI:** 10.1371/journal.pone.0066284

**Published:** 2013-06-04

**Authors:** Laetitia Delort, Charlotte Lequeux, Virginie Dubois, Alice Dubouloz, Hermine Billard, Ali Mojallal, Odile Damour, Marie-Paule Vasson, Florence Caldefie-Chézet

**Affiliations:** 1 Clermont Université, Université d'Auvergne, UFR Pharmacie, Laboratoire SVFp, Clermont-Ferrand, France; 2 INRA, UMR 1019, ECRIN, CRNH Auvergne, Clermont-Ferrand, France; 3 Centre Jean Perrin, Unité de Nutrition, Clermont-Ferrand, France; 4 Banque de tissus et de cellules, Hôpital Edouard-Herriot, Lyon, France; 5 Service de chirurgie plastique, reconstructrice et esthétique, Hôpital Edouard-Herriot, Lyon, France; University of Nebraska Medical Center, United States of America

## Abstract

Breast cancer has become the most common cancer among women in industrialized countries. Obesity is well established as a risk factor, in particular owing to the attendant secretion of the entities called adipokines; there is growing evidence for a role of cells and factors present in the mammary tumor microenvironment such as fibroblasts, preadipocytes, adipocytes and their secretions. To study how the microenvironment influences breast cancer growth, we developed a novel tridimensional adipose model epithelialized with normal human keratinocytes or with breast cancer cell lines. These mimicked a breast tumor in contact with an adipose microenvironment and allowed monitoring of the interactions between the cells. Leptin and adiponectin, two major adipokines, and their respective receptors, ObRt and AdipoR1, were expressed in the model, but not the second adiponectin receptor, AdipoR2. The differentiation of preadipocytes into adipocytes was greater when they were in contact with the breast cancer cell lines. The contact of breast cancer cell lines with the microenvironment completely modified their transcriptional programs by increasing the expression of genes involved in cell proliferation (*cyclinD1*, *MAPK*), angiogenesis (*MMP9*, *VEGF*) and hormonal pathways (*ESR1*, *IL6*). This tridimensional adipose model provides new insights into the interactions between breast cancer cells and their adipose microenvironment, and provides a tool to develop new drugs for the treatment of both cancer and obesity.

## Introduction

Breast cancer is by far the most common cancer among women worldwide, with 1.38 million new cases diagnosed in 2008, causing 458,000 deaths. The incidence of this cancer varies geographically being low in developing regions (South America, Africa and Asia) and a high incidence in developed countries (US and Western Europe) [1].

Obesity is now recognized as a risk factor for breast cancer, particularly in postmenopausal women, and is correlated with poor prognosis, larger tumor size, a higher incidence of lymph node metastasis and a high tumor grade [2]. In 2005, the world overweight population was 937 million (23.2%), of whom 396 million were obese (9.8%). In many industrialized countries, over one fifth of the adult population is obese, and this proportion is also increasing in developing countries [3].

Understanding the role of adipose tissue in breast cancer growth is of major interest in obese patients. There are several possible explanations of the relationship between breast cancer and adiposity. First, the larger amount of adipose tissue in obese patients is associated with a higher conversion of androgens to estrogens by aromatase, and higher circulating levels of insulin and insulin-growth factor (IGF), which then enhance cell proliferation [4]. Second, adipose tissue can produce greater amounts of the entities called adipokines, whose concentrations are modulated by obesity. Some recent studies have shown that adipose tissue is not only fat storage tissue, but is also an endocrine organ. We have demonstrated that leptin and adiponectin, the two main adipokines studied, exert proliferative and antiproliferative activities respectively on breast cancer cells *in vitro* [5]. Leptin, whose serum concentration is increased in obesity and breast cancer [6], stimulates cell proliferation by up-regulating the expression of cdk2 and cyclinD1 [7]. Inversely, low levels of adiponectin are significantly associated with an increased risk of breast cancer [8], [9]. However, *in vitro* assays have shown that adiponectin decreased the proliferation of breast cancer cell lines (MCF7, MDA-MB-231) and induced apoptosis and cell cycle arrest [10], [11]. In addition, these two adipokines and their respective receptors (ObR, AdipoR1 and AdipoR2) are detected by immunohistochemistry in *in situ* carcinomas, invasive ductal carcinomas and healthy adjacent tissues [5].

New hypotheses concerning the role of the total local tumor microenvironment have recently emerged. Mammary epithelial cells are surrounded by stromal cells, such as preadipocytes, adipocytes, fibroblasts, and endothelial and inflammatory cells, and there is increasing evidence that factors produced by this tumor microenvironment, including extracellular matrix components and direct cell-cell contact, impact on breast carcinogenesis [12]. For example, adipocytes may promote cancer progression [13], and endothelial cells may regulate cancer cell proliferation and invasiveness. A recent study showed that the secretions of “dysfunctional” endothelial cells could enhance hyperplasia, angiogenesis and inflammation [14]. Variations in fibroblast distribution can also influence local breast estrogen synthesis and thereby promote tumor growth [15].

The role of this microenvironment in the proliferation of healthy or malignant cells is still not clear but warrants investigation in the case of breast cancer. We devised a tridimensional (3D) model to study the interactions between the adipose tumor microenvironment and breast cancer cells. For this purpose, an innovative adipose 3D model epithelialized with normal human keratinocytes or with breast cancer cell lines was developed. The latter mimicked a breast tumor, allowing a direct contact with the adipose microenvironment. A comparison of these models showed interactions in both directions: breast cancer cells were able to act on adipocyte differentiation, and the adipose microenvironment strikingly modified breast cancer cell transcriptional programs involved in major biological pathways.

## Materials and Methods

### Cell cultures

Primary cultures of keratinocytes and fibroblasts were established from human skin obtained from patients undergoing surgery, in accordance to ethical and safety guidelines drawn up in French regulation n° DC-2008-162.

*Fibroblasts* were isolated from the foreskin of a child donor (age<10 years). They were grown in Dulbecco's modified Eagle's medium (DMEM with glutamax-1, Invitrogen, Cergy-Pontoise, France) supplemented with 10% calf serum (HyClone, Logan, Utah, USA), 20 µg/ml gentamicin (Pantapharm, Fougères, France), 100 UI/ml penicillin (Sarbach, Suresnes, France) and 1 µg/ml amphotericin B (Bristol Myers Squibb, Puteaux, France).

*Keratinocytes* were obtained from the foreskin of a child donor (age<10 years). They were grown in a 3∶1 mixture of DMEM and HAM F12 (Invitrogen), supplemented with 10% calf serum (HyClone), 10 ng/ml epidermal growth factor (EGF) (Austral biologic, San Ramon, California, USA), 0.12 UI/ml insulin (Lillly, Saint-Cloud, France), 0.4 µg/ml hydrocortisone (Up John, Saint-Quentin-en-Yvelines, France), 5 µg/ml triiodo-l-thyronine (Sigma, Saint-Quentin-Fallavier, France), 24.3 µg/ml adenine (Sigma) and antibiotics as above. All the skin samples were obtained with informed consent of their donors.

*Human Adipose derived Stem Cells (ASC) from adipose tissue* were isolated from lipoaspiration of donors undergoing optimized liposuction [16] using a 3 mm canula according to ethical and safety guidelines as approved by the local IRB and as described by Björntorp [17]. Briefly, adipose tissue was digested with collagenase (0.120 U/ml, Roche, Indianapolis, USA) at 37°C for 30 min and under constant shaking. Digestion was stopped by adding Dulbecco's modified Eagle's medium (DMEM with glutamax, Gibco (Invitrogen, Carlsbad, USA)) containing 10% fetal calf serum (FCS, HyClone, Logan, USA). Floating adipocytes were discarded and cells from the stromal-vascular fraction (SVF) were pelleted, rinsed with media, centrifuged (300 gmin^−1^ for 5 min, 20°C) and incubated in an erythrocyte lysis buffer for 10 min at 37°C. This cell suspension was centrifuged (300 g.min^−1^ for 5 min, 20°C) and cells were counted using trypan blue and seeded at a density of 8×10^4^ cells/cm^2^.

*The mammary cell lines MCF7, MDA-MB-231 and MCF10a* were obtained from the American Type Culture Collection (ATCC). MCF7 cells, which possesses estrogen receptor α (ER+), were cultured in RPMI 1640 medium (Pan Biotech, France) supplemented with 10% heat-inactivated fetal bovine serum (FBS, Abcys, France), 0.04 U/mL insulin, 50 µg/mL gentamycin and 2 mM l-glutamine (Pan Biotech, France) and incubated at 37°C in a humidified atmosphere containing 5% CO_2_. MDA-MB-231 cells, which are non-estrogen dependent cells and more invasive than MCF7, were grown in L15 Leibovitz (Pan Biotech, France) supplemented with 15% FBS, 50 µg/mL gentamycin and 2 mM l-glutamine and incubated at 37°C in a humidified atmosphere. MCF10a cells were cultured in DMEM/F-12 (Pan Biotech, France) supplemented with 10% horse serum (Pan Biotech, France), 20 ng/ml epidermal growth factor (EGF), 0.25 U/mL insulin, 0.5 µg/ml hydrocortisone (Sigma, Saint-Quentin-Fallavier, France), 100 ng/ml cholera toxin (Sigma, Saint-Quentin-Fallavier, France) and 50 µg/mL gentamycin in a 5% CO_2_-humidified incubator at 37°C

### 3D Culture

**Figure 1:**
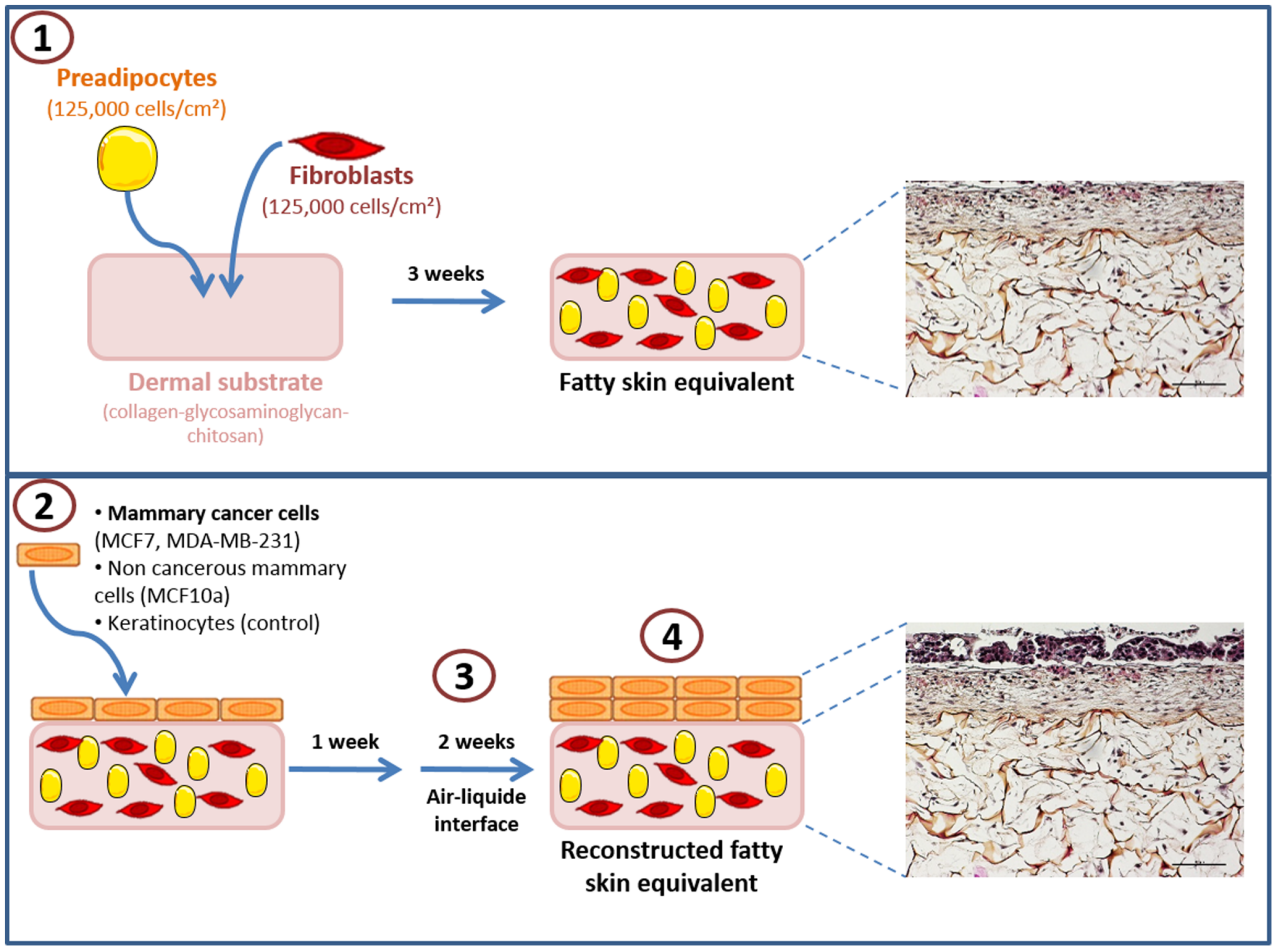
Development of the adipose skin equivalent. 1. Fibroblasts and preadipocytes were seeded on a dermal substrate. 2. After 3 weeks of culture, a fatty equivalent dermis was obtained and keratinocytes (control) or mammary cancer cells (MCF7, MDA-MB-231) or non-cancerous mammary cells (MCF10a) were then seeded on the dermis. 3. After one week of culture, these cells were grown at the air-liquid interface (2 weeks) to obtain the reconstructed fatty skin equivalent. 4. At the end of the experimentation, epithelial cells (*n* = 3) were separated from the dermis with a thermolysin treatment to extract RNA. The expression of 32 key genes was investigated by qRT-PCR and compared with the expression of cells cultured in normal conditions.

We used a 3D model of skin equivalent [18], [19] (Patent PCT/FR/8800303, 1989) replacing the skin equivalent by an adipose skin equivalent in which breast cancer cell lines were in contact with different cell types such as fibroblasts, preadipocytes and adipocytes (Figure 1). This 3D structure mimicked a breast tumor surrounded by a microenvironment, with breast cell lines being in contact with fibroblasts, preadipocytes and mature adipocytes, and with all their secretions.

Fibroblasts (125,000 cells/cm^2^) and preadipocytes (125,000 cells/cm^2^) were seeded on top of the collagen-glycosaminoglycan-chitosan porous scaffold (dermal substrate) and cultured in DMEM/HAM F-12 (1∶1) supplemented with 10% FBS, *basic*FGF (10 ng/mL), vitamin C and streptomycin (50 µg/mL), which was replenished every 2 days. After 3 weeks of culture, a fatty equivalent dermis was obtained and four different epithelial cell lines (one million) were seeded on the surface: keratinocytes (skin equivalent control), MCF10a, MCF7, and MDA-MB-231. Three independent specimens of each studied mammary cell line were used. At this step, the medium is a mixture between keratinocyte medium [20] and an optimized adipocyte differentiation medium [19]. Briefly it contains DMEM/HAM F12 (1∶1) supplemented with 10% FBS, hydrocortisone, insulin, adenine, EGF, T3, vitamin C, dexamethazon, roziglitazone, IBMX (only the first three days), streptomycin and fungizon [19]. The medium was replenished every 2 days. After 1 week of culture, cells were grown at the air-liquid interface for 2 weeks to obtain the reconstructed fatty skin equivalent, *i.e.* 3D adipose skin equivalent.

### Histological analysis

3D adipose skin equivalent was fixed in 10% buffered formalin and embedded in paraffin. Tissue sections 5 µm thick were stained with hematoxylin phloxin safran (HPS) to visualize nucleus, cytoplasm and ECM formation.

### Oil Red O staining for evaluation of adipose differentiation

All the 3D adipose skin equivalents were fixed in O.C.T compound (TISSUE-TECK, Sakura, Netherlands) and frozen at −20°C. They were then cut into 5 µm-thick sections, stained with Oil Red O (5 mg/ml, Sigma) and counterstained with Harris hematoxylin (Sigma).

### Immunohistochemistry

3D adipose skin equivalent was fixed with formol and embedded in paraffin wax. Sections 0.5 µm thick were cut. The procedure was as described previously [21], [22]. Briefly, sections were deparaffinized and rehydrated in graded alcohol and distilled water. Slides were pretreated by boiling in citrate buffer for 40 minutes at pH = 6. The avidin/biotin kit (Vector Laboratories, Burlingame, USA) was used to block non-specific binding sites. Leptin, adiponectin, AdipoR1, AdipoR2, and Ob-Rt protein expression was investigated by immunohistochemical staining using affinity-purified polyclonal biotinylated antibodies raised against leptin, Ob-Rt (corresponding to the extracellular domain recognizing all six forms of Ob-R), adiponectin (R&D Systems, France), AdipoR1, AdipoR2 (Phoenix Pharmaceuticals, France) (1 µg/mL) diluted in PBS-SAB 5% overnight at 4°C in a humidified chamber.

Antigen staining was performed using ABC peroxidase-conjugated streptavidin kit for 30 min and the specimens treated with DAB substrate for 10 minutes (Vector Laboratories, Burlingame, USA). Sections were then contrasted using hematoxylin, dehydrated and mounted using the Vectastain mounting medium.

### Molecular analysis

Epithelial cells were separated from the rest of the skin by treatment with thermolysin and immediately frozen on liquid nitrogen. Total RNA was extracted by grinding in nitrogen and with Trizol according to the manufacturer's recommendations (Invitrogen). The quantity and quality of RNA was assessed by 260/280 ratio using a NanoDrop 8000 spectrophotometer (Thermo Fisher Scientific). cDNA was obtained with HighCap cDNA RT Kit RNAse inhib (Applied Biosystems).

Relative expression of 32 genes of interest was assessed by Taqman Express plates on ABI PRISM 7900 HT (Applied Biosystems). Among the selected genes (Table 1), we determined the expression of genes coding for the two major adipokines (leptin and adiponectin) and their receptors (ObR, AdipoR1 and AdipoR2), genes involved in hormonal pathway (estrogen receptors, progesterone receptor, aromatase, insulin, IGF1), and genes coding for cytokines (IL6, TNF). We also determined the expression of genes involved in proliferation (AKT, MAPK1, cyclin D1), in apoptosis (Bax, Bcl2), in angiogenesis (MMP2, MMP9, VEGF), the expression of transcription factors (PPARα, PPARγ, STAT3), tumor suppressor genes (BRCA1, TP53, e-cadherin) and oncogene (MYC).

**Table 1:** List of the selected genes.

Gene symbol	Gene name	Assay reference
18S	Eukaryotic 18S rRNA	18S-Hs99999901_s1
TP53	tumor protein p53	TP53-Hs01034249_m1
BAX	BCL2-associated X protein	BAX-Hs00180269_m1
BCL2	B-cell CLL/lymphoma 2	BCL2-Hs99999018_m1
CCND1	cyclin D1	CCND1-Hs99999004_m1
MYC	v-myc myelocytomatosis viral oncogene homolog	MYC-Hs00905030_m1
IGF1	insulin-like growth factor 1	IGF1-Hs00153126_m1
IGF1R	insulin-like growth factor 1 receptor	IGF1R-Hs99999020_m1
LEPR	leptin receptor	LEPR-Hs00174492_m1
ADIPOR1	adiponectin receptor 1	ADIPOR1-Hs01114951_m1
ADIPOR2	adiponectin receptor 2	ADIPOR2-Hs00226105_m1
ADIPOQ	adiponectin	ADIPOQ-Hs00605917_m1
LEP	leptin	LEP-Hs00174877_m1
INS	insulin	INS-Hs00355773_m1
INSR	insulin receptor	INSR-Hs00961560_m1
ESR1	estrogen receptor 1	ESR1-Hs01046812_m1
ESR2	estrogen receptor 2 (ER beta)	ESR2-Hs01100356_m1
PGR	progesterone receptor	PGR-Hs01556707_m1
CYP19A1	cytochrome P450, family 19, subfamily A, polypeptide 1	CYP19A1-Hs00240671_m1
VEGFA	vascular endothelial growth factor A	VEGFA-Hs00173626_m1
CDH1	cadherin 1	CDH1-Hs00170423_m1
MMP2	matrix metallopeptidase 2	MMP2-Hs00234422_m1
MMP9	matrix metallopeptidase 9	MMP9-Hs00957562_m1
STAT3	signal transducer and activator of transcription 3	STAT3-Hs01047580_m1
AKT1	v-akt murine thymoma viral oncogene homolog 1	AKT1-Hs00920503_m1
MAPK1	mitogen-activated protein kinase 1	MAPK1-Hs01046830_m1
TNF	tumor necrosis factor	TNF-Hs00174128_m1
IL6	interleukin 6	IL6-Hs00174131_m1
PPARA	peroxisome proliferator-activated receptor alpha	PPARA-Hs00947539_m1
PPARG	peroxisome proliferator-activated receptor gamma	PPARG-Hs01115513_m1
BRCA1	breast cancer 1, early onset	BRCA1-Hs01556194_m1
GAPDH	glyceraldehyde-3-phosphate dehydrogenase	GAPDH-Hs99999905_m1

The PCRs were performed in 96-well plates in a total volume of 20 µL and 30 ng of cDNA. The program was as follows: two initial steps at 50°C for 2 min and 95°C for 10 min, and then 40 cycles of 95°C for 15 s and 60°C for 60 s.

Relative amount was calculated from the threshold cycles with the instrument's software (SDS 2.0) according to the manufacturer's instructions. Genes were considered significantly expressed and their transcript measurable if their corresponding Ct value was less than or equal to 35. The expression levels of the target genes in the reconstructed skin fatty equivalent were compared with the expression of the same cell lines cultured under classic conditions, *i.e.* in their respective culture medium in flasks. The comparative cycle threshold (CT) method (2^−ΔΔCT^) was used to calculate the relative gene expression of a given sample in 3D skin equivalent, normalized within the sample to an endogenous reference gene (GAPDH), and relative to the expression of the same gene in another sample (cell cultured alone): 2^−ΔΔCT^ method with ΔΔCT = [ΔCT (3D skin equivalent) - ΔCT (cell cultured alone)] and ΔCT = [CT(target gene) - CT(reference gene)]. Student's *t* test was used for comparisons of gene expression levels in cells cultured in 3D and in cells cultured alone.

### Statistical analysis

Principal component analysis (PCA), an unsupervised multivariate analysis, was carried out to explore the differences among samples and relationships among genes. Hierarchical cluster analysis (HCA) was used with Euclidian distance and Ward aggregation to classify samples and genes. Both techniques were performed with unit variance scaling on gene expression data.

For PCA, we used SIMCA-P+ v12 software (Umetrics, Umea, Sweden) and for HCA Permutmatrix v1.93 [23].

## Results

### Reconstructed three-dimensional (3D) adipose equivalent-Differentiation of preadipocytes

**Figure 2:**
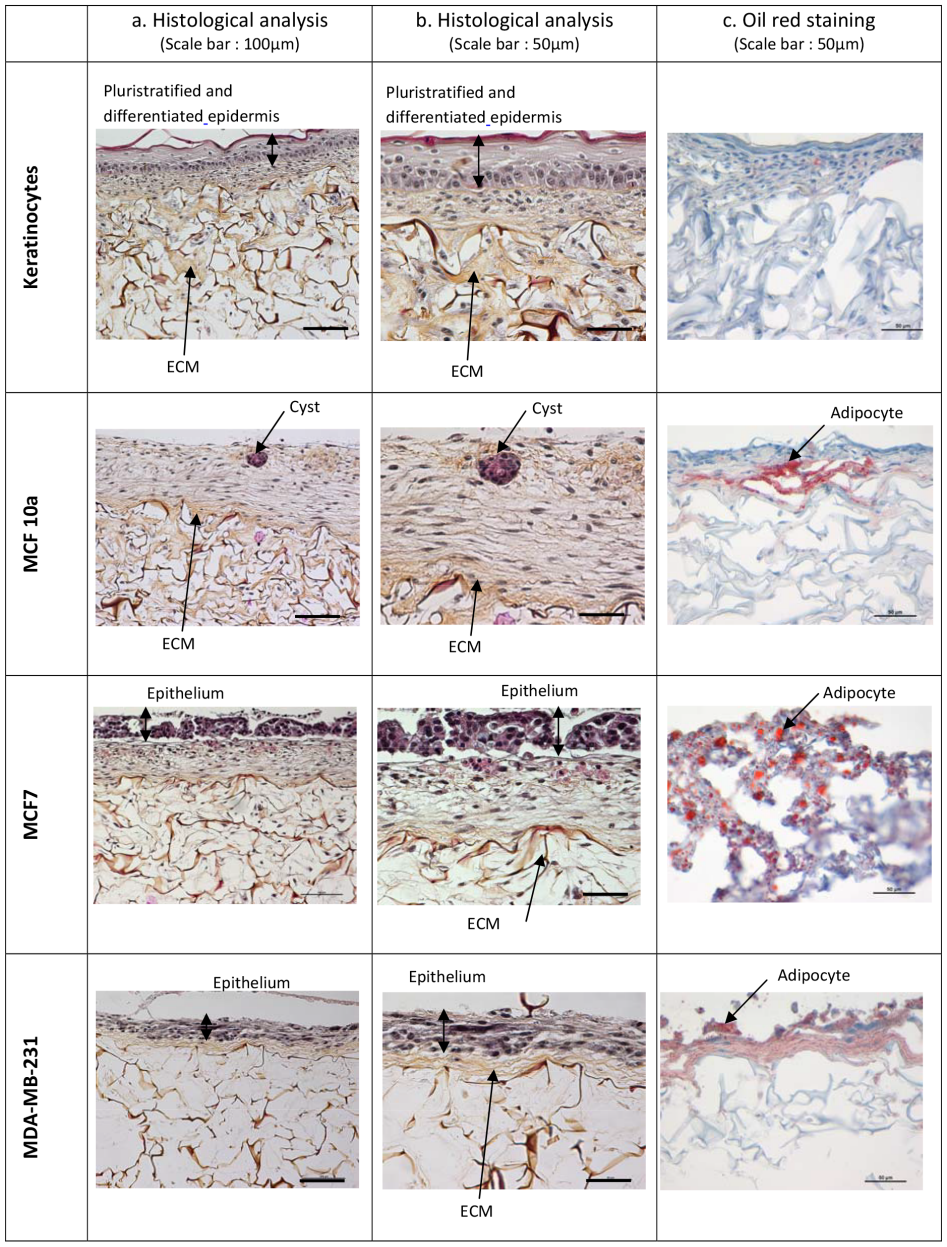
Influence of epithelial cells on adipose connective tissue: a) and b). Hematoxylin Phloxin Safran (HPS) staining for nucleus (purple), cytoplasm (red) and extracellular matrix components (orange); c. Oil red staining: mature adipocytes appeared in red.

The experiment was validated with the results of culture control. Indeed, the 3D adipose model obtained with normal human keratinocytes (NHK) exhibited a balanced, pluristratified, and differentiated epidermis, close to human normal skin (Figure 2). HPS staining showed that fibroblasts and preadipocytes colonized the dermal substrate. The negative Oil Red O staining specific to mature adipocytes showed that no differentiation of preadipocytes into mature adipocytes occurred.

In, contrast to the NHK model, the differentiation of preadipocyte to mature adipocytes was observed in our 3D model with breast epithelial cells. In addition, differences were observed according to the cell line. With cystic mammary cells (MCF10a cell line), cysts were present in the epithelium and with cancerous MCF7 and MDA-MB-231 cell lines, the epithelium appeared pluristratified but non-differentiated and completely disorganized. Deeper in adipose connective tissue, preadipocyte differentiation into mature adipocytes was greater with these two cancerous cell lines. These results suggest that preadipocyte differentiation into mature adipocytes was only possible in the presence of breast cells in their microenvironment with a greatest effect occurring with breast cancer cells. They also suggest that interactions take place between adipose and cancerous cells as a result of the secretion of biomolecules through mechanisms not yet fully understood.

### Breast cancer cells expressed adipokines and their receptors except for AdipoR2

**Figure 3:**
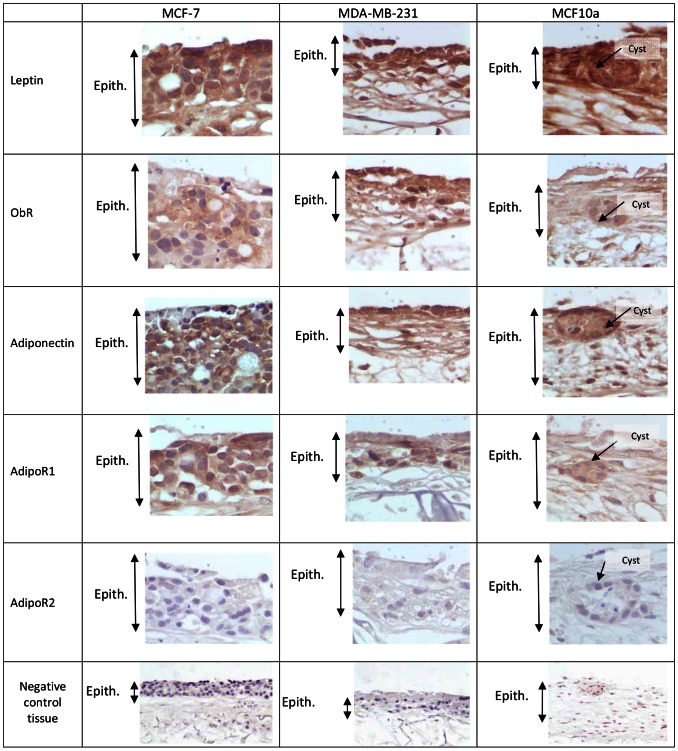
Immunohistochemical staining of the tridimensional adipose skin equivalent using affinity-purified polyclonal biotinylated antibodies raised against Leptin, Ob-Rt, Adiponectin, AdipoR1, AdipoR2. (Manification: ×400). Positive staining appears in brown. (Epith: Epithelium)

The two major adipokines, leptin and adiponectin, were strongly expressed in all three models whatever the mammary epithelial cells used, cancerous MCF7, MDA-MD-231 and non-cancerous mammary cells MCF10a (Figure 3), but not in the model epithelialized with NHK. Their respective receptors, ObRt and AdipoR1 were expressed to a lesser extent. Unexpectedly, AdipoR2 was not expressed.

### Adipose microenvironment modified the expression of genes involved in major biological pathways

Quantitative real-time PCR was used to assess the expression of 32 genes coding for adipokines and their receptors, for proteins involved in hormonal pathways, and in major biological pathways such as proliferation and angiogenesis, and to compare breast epithelial cells cultured alone in monolayered culture and cells in 3D adipose connective tissue model. The presence of fibroblasts and adipose cells (preadipocytes and mature adipocytes) and their secretions strikingly modified gene expression in breast cells.

**Figure 4:**
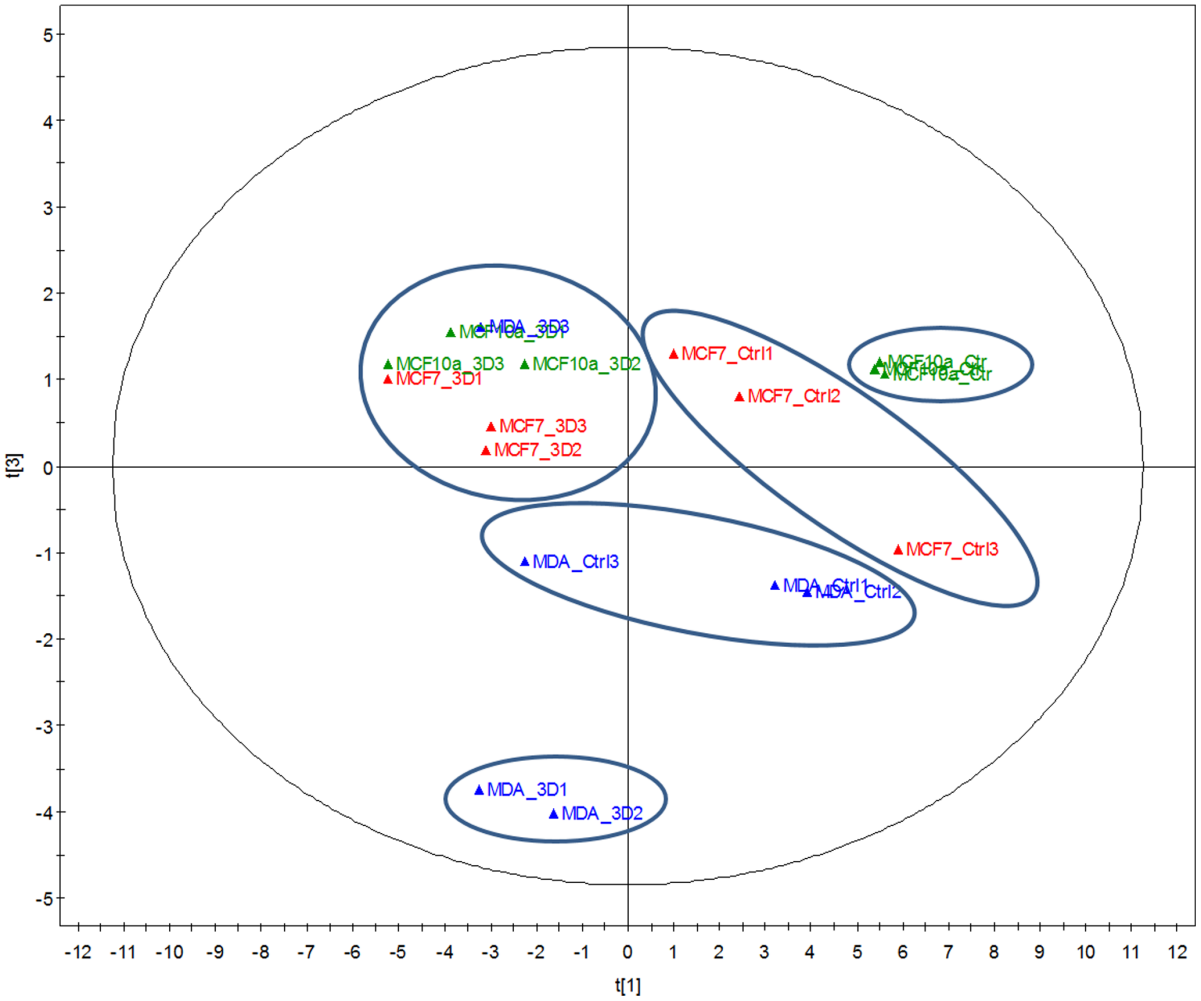
Principal component analysis (PCA) carried out to explore the differences among samples and relationships among genes. The expression of genes by mammary cell lines (MCF7, MDA-MB-231) cultured in control conditions (MCF7_Ctrl, MDA_Ctrl, MCF10a_Ctrl) or cultured in the adipose skin equivalent model (MCF7_3D, MDA_3D, MCF10a_3D) was evaluated using unit variance scaling on gene expression data. On the first principal component, a clear discrimination can be noted between cells cultured in the adipose skin equivalent model versus cells cultured in normal conditions. The second principal component permitted to discriminate the three studied cell lines (MCF7, MDA-MB-231 and MCF10a) among the control group.

The analysis of gene expression was made by principal component analysis (PCA) (Figure 4). The first two principal components account for 63% of the total variance. On the first principal component, a clear discrimination can be seen between cells cultured in the adipose skin equivalent model and those cultured in normal conditions. The second principal component discriminated the three studied cell lines (MCF7, MDA-MB-231 and MCF10a) in the control group. In contrast, PCA showed that the cell lines cultured in the adipose skin equivalent model tended to exhibit the same expression profile. However, two assays of the MDA-MB-231 cells cultured in the 3D model seemed to be at distance from this plot.

**Figure 5:**
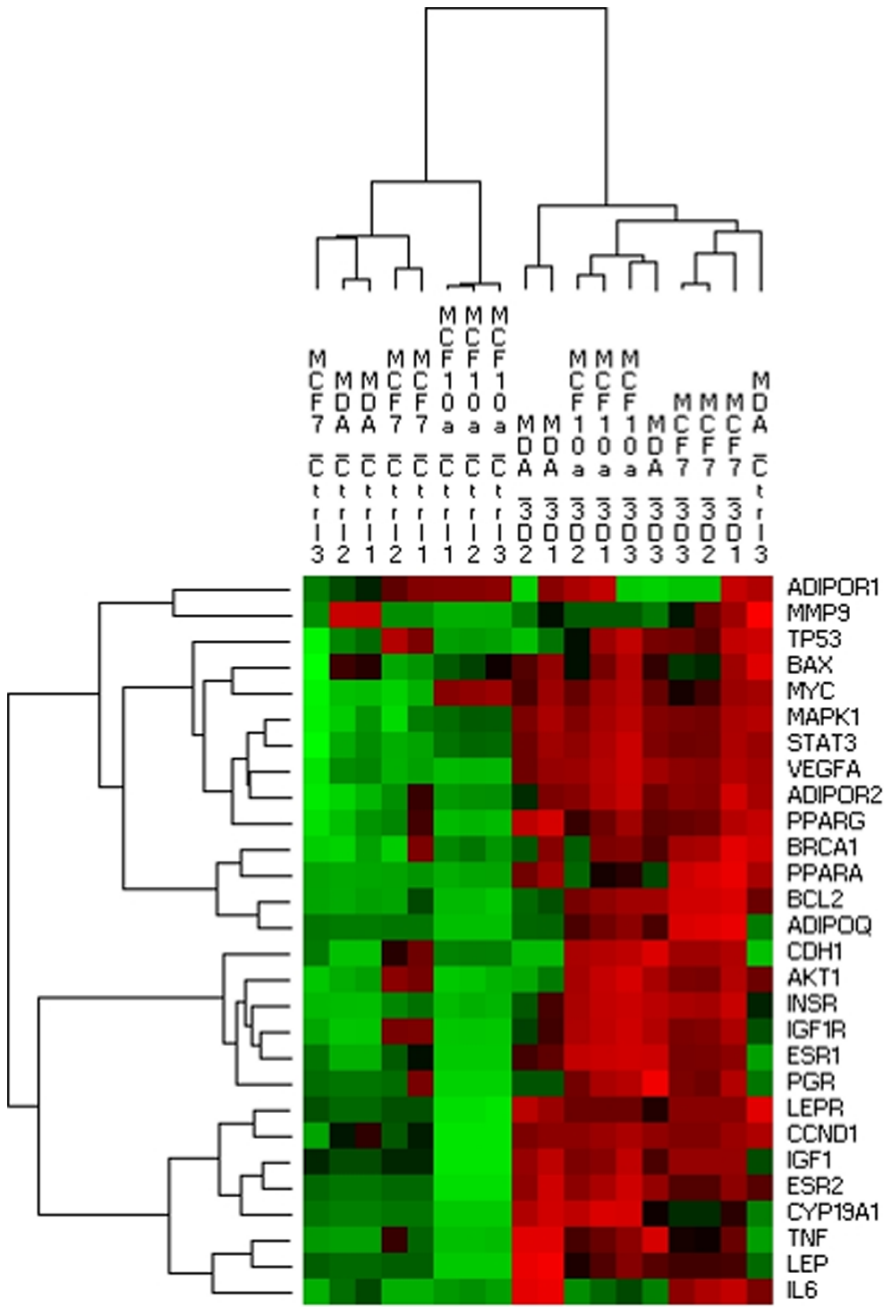
Hierarchical cluster analysis of the genes differentially expressed between the three studied mammary cell lines (MCF7, MDA-MB-231, MCF10a) in control conditions (Ctrl) or cultured in the adipose skin adipose model (3D). Expression blocks were formed, the red block being more expressed than the green block.

Hierarchical cluster analysis (HCA) (Figure 5) allowed us to evaluate the low to high expression of each gene in each control and 3D assay. It discriminated cells cultured in control conditions and cells cultured in the 3D model, except for one assay of MDA-MB-231 cultured in control condition. This representation led to the formation of expression blocks, red and green, of which the former was more strongly expressed.

The relative gene expression of a given sample in the 3D skin equivalent model calculated with the 2^−ΔΔCT^ method (Table 2), revealed significant modifications in genes coding for adipokines in MCF10a, with a 10-fold up regulation of leptin gene, 23-fold for adiponectin and 5-fold for AdipoR2. Up-regulation of *ESR1* was observed in MCF7 and MDA-MB-231 and that of *ESR2* in MCF7 and MCF10a. The expression of aromatase was unchanged irrespective of cell type. There was a marked increase in *TNF* and *IGF1* in MCF10a, and an increase in *INSR* in all three cell lines.

**Table 2:** Variation of gene expression in the different cell lines studied between.

	MCF7		MDA-MB-231		MCF10A	
	Expression ratio^a^	*P* value^b^	Expression ratio	*P* value	Expression ratio	*P* value
***Adipokines***						
LEP	-		14.2	0.10	**10.3**	**0.01**
LEPR	-		1.2	0.82	-	
Adiponectin	-		1.9	0.19	**23.3**	**0.01**
ADIPOR1	0.4	0.52	0.1	0.37	3.5	0.74
ADIPOR2	4.8	0.06	2.2	0.34	**5.1**	**0.02**
***Hormonal pathway, cytokines***						
CYP19A1	1.9	0.01	8.3	0.09	-	
ESR1	**16.1**	**0.01**	**281.6**	**0.04**	-	
ESR2	**2.1**	**0.03**	3.3	0.11	**18.6**	**0.01**
PGR	**2.5**	**0.01**	3.9	0.33	**21.9**	**0.01**
IGF1	-		2.0	0.09	**9.2**	**0.01**
IGF1R	2.7	0.23				
IL6	**44.4**	**0.01**	10.3	0.39	2.2	0.19
INS	-		-		-	
INSR	**18.8**	**0.01**	6.8	0.01	48.9	0.01
TNF	2.2	0.25			17.9	0.01
Angiogenesis						
MMP2	-		-		-	
MMP9	3.2	0.05	0.1	0.07	-	
VEGFA	15.9	0.02	2.9	0.19	21.8	0.01
Apoptosis						
BAX	3.5	0.11	0.8	0.69	1.7	0.14
BCL2	68.7	0.01	2.8	0.01	41.3	0.01
Proliferation-survival						
AKT1	2.2	0.32	1.4	0.15	-	
MAPK1	11.3	0.03	3.2	0.32	4.2	0.03
CCND1	9.1	0.04	1.7	0.48	-	
Transcription factors						
PPARA			2.5	0.53	3.6	**0.03**
PPARG	3.9	0.08	3.4	0.39	5.3	**0.02**
STAT3	11.4	0.06	2.6	0.26	5.2	**0.02**
Tumor suppressor genes, oncogenes						
BRCA1	7.1	0.02	1.6	0.63	2.7	0.13
TP53	1.7	0.48	0.7	0.17	2.8	0.06
CDH1	13.4	0.05	23.2	0.39	-	
MYC	7.0	0.01	2.6	0.33	1.2	0.81

a Average expression level in 3D skin equivalent calculated with the 2^−ΔΔCT^ method.

b *P* values were obtained using a two-tailed Student's *t* test to identify genes whose average expression levels were statistically significantly different between 3D skin equivalent and cells cultured alone.

Induction of *Cyclin D1* (9-fold in MCF7) and *MAPK* (11-fold in MCF7 and 4.2-fold in MCF10a) was observed. Key changes also involved the induction of tumor suppressor genes such as *BRCA1* (7-fold), *CDH1* (13-fold), and oncogenes (*MYC*: 7-fold) in MCF7 cells, and the induction of transcription factors in MCF10a (*PPARα*: 3.6-fold; *PPARβ*: 5.3-fold; *STAT3*: 5.2-fold).

Anti-apoptotic *Bcl2* gene was consistently up-regulated (68-fold in MCF7; 2.8-fold in MDA-MB-231; 41.3 in MCF10a), and the expression of *Bax* was not modified.

The angiogenesis pathway was activated with a 3-fold increase for *MMP9,* a 15-fold increase for *VEGF* in MCF7 and a 21-fold increase for *VEGF* in MCF10a, but was not modified in MDA-MB-231 cells.

## Discussion

To gain a fuller understanding of the role of the adipose microenvironment in breast cancer growth, we developed a novel model of reconstructed adipose connective tissue that could be epithelialized either with cutaneous NHK, to produce a skin equivalent, or with cystic or tumor breast epithelial cells, to allow interactions between these breast epithelial cells and different cell types (fibroblasts, adipose cells and their secretions). Three mammary cell lines were studied in our model: MCF10a cells derived from a fibrocystic disease, considered as non-tumorigenic, served as a breast cell control, and two cancer cell lines, MCF7, which possesses estrogen receptor α (ER+), and MDA-MB-231, estrogen-insensitive cells which are considered to be more invasive. We observed a stronger differentiation of adipocytes in contact with cancer cells, and marked modifications of mammary cell transcriptional programs, which suggests the existence of reciprocal interactions.

Breast tumors are surrounded by different cell types including endothelial cells, fibroblasts, preadipocytes and adipocytes, and each cell type has the ability to secrete biomolecules that can act on breast cancer cells. Adipose tissue is now considered as an endocrine organ, in addition to being a fat storage tissue. It secretes several entities called adipokines, the most widely studied of which are leptin and adiponectin, which are respectively increased and decreased in obese patients [24]. Leptin is produced by preadipocytes, adipocytes, mammary epithelial cells and other tissues [25]. Increased serum concentration of leptin is correlated with obesity, and a high level of leptin is associated with breast cancer risk [6]. This adipokine is known to exert its activity through its receptor ObR, and ObR mRNA and their protein expressions have been observed in breast cancer cell lines [7]. Adiponectin, considered as a protective factor because it decreases the proliferation of cancerous cells [7], is produced sparingly by cancerous mammary cells but abundantly by adipose cells. Also, lower serum adiponectin concentrations have been reported in obese patients and in menopausal women with breast cancer [26].

The high protein expression of leptin and its receptor and the surprising absence of AdipoR2 in the adipose skin equivalent seeded with normal human keratinocytes point to the possible role of an adipose microenvironment in increasing leptin proliferative activity and down-regulating the expression of adiponectin receptor. Immunohistochemical studies have shown that leptin, adiponectin and their receptors (Ob-R, AdipoR1 and AdipoR2) are detected in the cytoplasm of MCF-7 breast cancer cells [5]. The mRNA of the two adiponectin receptors were also found in MDA-MB-231 [27]. Immunoblotting of total protein lysates showed that the two major isoforms of Ob-R were expressed at similar levels in different cell lines including MCF-7 and MDA-MB-231 [28]. MCF10a cells expressed leptin [29]. These results are in agreement with studies conducted on human biopsies showing leptin and Ob-R protein expression in breast cancer cells and in benign breast lesions [30].

Cancer cells (MCF-7 and MDA-MB-231) expressed adipokines at the transcriptional level. Nevertheless, no significant increase in these expressions was observed in the presence of the adipose microenvironment. The transcriptional levels of adiponectin MCF10a cells were significantly higher in the 3D configuration, which could explain their benign character compared to invasive MCF-7 cells.

We suggest there is a cross-talk between cancerous and adipose cells that allows maximal differentiation of preadipocytes into mature adipocytes, irrespective of estrogen receptor status. The same process was reported by Wang et al., who observed that tumor invasive potential was only obtained when these cells were cultured in a medium conditioned by both murine adipose cells and human breast cancer cells [13]. Inversely, media conditioned by MCF7 and MDA-MB-231 induced a reversion of human and murine adipose cells, the main component of the breast stroma, to a fibroblastic phenotype *via* two soluble factors (TGF-β1 and TNF-α). Also, adipocytes cultured with this conditioned media presented a decreased lipid accumulation [31]. Thus tumor cells may be able to modulate their microenvironment to favor tumor progression.

Estrogens are key biological factors in promoting breast carcinogenesis in estrogen receptor tumors. Adipose tissue becomes the most abundant producer of estrogen after menopause, as a result of aromatization of androgens by the aromatase. Our model shows differences in the hormonal pathways modified by the tumor environment. By contrast, the differentiation of mature adipocytes was not affected by estrogen receptor status. We postulate that (i) fibroblasts in contact with breast cells have higher aromatase activity, thereby enhancing estrogen biosynthesis, which in turn favors the progression of breast cancer [15], and (ii) although aromatase expression in breast cancer cells was not increased in our model, other markers that are able to stimulate aromatase activity, such as *IL6*, *TNFα*, *IGF-1,* exhibited greater mRNA expression [32]. Hence, the secretions of fibroblasts and adipocytes could result in higher estrogen production and in turn could increase breast cancer proliferation by interacting with estrogen receptors on surrounding cancer cells in a paracrine manner.

Increased BMI in postmenopausal women is associated with hyperinsulinemia, insulin-resistant type 2 diabetes and metabolic syndrome, which in turn are associated with a small increase in hormonal breast cancer risk. These conditions lead to elevated glucose blood concentration, increased secretion of insulin and induction of ER+ breast cancer cell lines [33]. The increase of INSR expression in our model suggested that estrogen and insulin cooperate to promote cell-cycle progression [34]. Also, insulin and IGF1 are able to stimulate ER transcriptional activity in breast cancer cells, even in the absence of estrogen [35].

Molecular analysis demonstrated that the tumor microenvironment induced profound changes in breast cancer (MCF7, ER+)and non-cancerous (MCF10a) cell proliferation. In contrast, the invasive character of MDA-MB-231 cells did not seem to be influenced by the adipose microenvironment. These results are in agreement with our previous observations (unpublished data) and those of Binai [36] who reported that leptin had no impact on proliferation of these estrogen-receptor negative cells. In addition, MDA-MB-231 cells often exhibited high basal gene expressions, which could also explain why gene expression ratio was unchanged.

The up-regulation of *VEGF* and matrix metalloproteinase 9, which have the ability to degrade the extracellular matrix, led to an increased invasive potential in MCF7 cancer cells. Proliferation was also stimulated with an increased expression of *MAPK1* and *cyclinD1* in these cells. The increase in *cyclin D1* expression could be explained by the production of biomolecules from the adipose microenvironment that could stimulate cyclin D1 such as leptin. Thus, invasive potential was notably increased in cells surrounded by adipose cells and their secretions, except for MDA-MB-231, which were initially the most invasive of the cells studied.

Conversely, an up-regulation of *CDH1* and *BRCA1* could lead to the suppression of cellular transformation. However, studies have shown the enhanced expression of E-cadherin can represent an early stage in ovarian cancer [37]. Genetic alterations were identified in tumor-associated stroma from several independent cases of mammary carcinomas, suggesting the role of these mutations in the formation of epithelial tumors [38].

Co-cultures of mammary cancer cells and mature adipocytes from rats showed that mature adipocytes unlike preadipocytes promoted the growth of ER-positive cancer cells, but no effect was observed on apoptosis. In addition, preadipocytes, but not mature adipocytes, promoted a higher expression of the E-cadherin, suggesting a protective role of this cell type by an increase in cell adhesion [39].

Our model show, for the first time, the existence of a cross-talk between human adipose microenvironment and breast cell lines: the adipose microenvironment seems to modify the expression profile of tumor cells and the tumor cells seem to influence the differentiation of mature adipocyte. Determining the specific role of adipokines in breast cancer cells is of major importance. The cancer microenvironment, including adipocytes and fibroblasts, must be considered globally to understand breast cancerogenesis, particularly in obese patients in whom the expression of adipokines and estrogen is modified.
